# CaMKIV-Mediated Phosphorylation Inactivates Freud-1/CC2D1A Repression for Calcium-Dependent 5-HT1A Receptor Gene Induction

**DOI:** 10.3390/ijms25116194

**Published:** 2024-06-04

**Authors:** Kimberly Galaraga, Anastasia Rogaeva, Nathan Biniam, Mireille Daigle, Paul R. Albert

**Affiliations:** Ottawa Hospital Research Institute (Neuroscience), Ottawa Brain and Mind Research Institute, 451 Smyth Road, Ottawa, ON K1H-8M5, Canada; kgalaraga@hotmail.com (K.G.); nist25@gmail.com (A.R.); nbiniam2@uottawa.ca (N.B.); mireille.daigle@uottawa.ca (M.D.)

**Keywords:** 5-HT1A receptor, transcription factor, raphe, phosphorylation, gene repression, major depressive disorder

## Abstract

Calcium calmodulin-dependent protein kinase (CaMK) mediates calcium-induced neural gene activation. CaMK also inhibits the non-syndromic intellectual disability gene, Freud-1/CC2D1A, a transcriptional repressor of human serotonin-1A (5-HT1A) and dopamine-D2 receptor genes. The altered expression of these Freud-1-regulated genes is implicated in mental illnesses such as major depression and schizophrenia. We hypothesized that Freud-1 is blocked by CaMK-induced phosphorylation. The incubation of purified Freud-1 with either CaMKIIα or CaMKIV increased Freud-1 phosphorylation that was partly prevented in Freud-1-Ser644Ala and Freud-1-Thr780Ala CaMK site mutants. In human SK-N-SH neuroblastoma cells, active CaMKIV induced the serine and threonine phosphorylation of Freud-1, and specifically increased Freud-1-Thr780 phosphorylation in transfected HEK-293 cells. The activation of purified CaMKIIα or CaMKIV reduced Freud-1 binding to its DNA element on the 5-HT1A and dopamine-D2 receptor genes. In SK-N-SH cells, active CaMKIV but not CaMKIIα blocked the Freud-1 repressor activity, while Freud-1 Ser644Ala, Thr780Ala or dual mutants were resistant to inhibition by activated CaMKIV or calcium mobilization. These results indicate that the Freud-1 repressor activity is blocked by CaMKIV-induced phosphorylation at Thr780, resulting in the up-regulation of the target genes, such as the 5-HT1A receptor gene. The CaMKIV-mediated inhibition of Freud-1 provides a novel de-repression mechanism to induce 5-HT1A receptor expression for the regulation of cognitive development, behavior and antidepressant response.

## 1. Introduction

Freud-1/CC2D1A was initially identified in a screening for transcriptional regulators of NF-kappaB [1] and is a transcription factor that has been implicated in neural plasticity, intellectual disability, anxiety and major depression [2,3]. Deletions or polymorphisms in the Freud-1 gene have been linked or associated with non-syndromal intellectual disability [4,5,6], and mice lacking Freud-1 globally or in forebrain glutamate neurons show altered dendritic development, cognitive impairment and anxiety, further indicating a role for Freud-1 in neural development and cognitive function [5,7,8]. In addition, Freud-1 represses the 5-HT1A receptor gene, a key regulator of the serotonin system and is implicated in anxiety, depression, and social interaction [7,9,10,11]. In humans, the 5-HT1A gene polymorphisms that alter the expression of 5-HT1A receptors and brain region specific changes in 5-HT1A receptor levels have been associated with depression and suicide [12,13,14]. In mice, the gene deletion of Freud-1 in 5-HT neurons during adulthood increases 5-HT1A autoreceptor expression, and is associated with fluoxetine-resistant depression and anxiety-like behaviors [10], while the knockdown of the 5-HT1A autoreceptor enhances the fluoxetine response [15,16]. Thus, elucidating the mechanisms of Freud-1 regulation could underlie the normal regulation of genes implicated in neural development and behavioral phenotypes.

We initially characterized Freud-1/CC2D1A as a calcium-regulated transcriptional repressor of the human and mouse 5-HT1A receptor genes [17,18], and of the human dopamine-D2 receptor gene [19]. Freud-1 contains four repeats of a conserved DM-14 domain of unknown function, a helix-loop-helix (HLH) region and a conserved C2 calcium phospholipid binding domain at the carboxyl-terminal portion [17,18]. The partial deletion of the C2 domain strongly reduced Freud-1 binding to its DNA element (dual repressor element, DRE) and inhibited its repressor activity at the 5-HT1A gene, suggesting a key role for the C2 or adjacent HLH domains in Freud-1 repression [17]. Calcium mobilization attenuated Freud-1 binding to and reduced Freud-1-mediated repression, which was reversed by the pharmacological inhibition of CaMK [17]. These findings suggested that the calcium-dependent induction of the 5-HT1A gene might involve the CaMK-induced inhibition of the Freud-1 repressor activity, but the mechanisms remain unclear. This study addresses the mechanism by which calcium inactivates Freud-1, leading to the activation of the 5-HT1A receptor gene [17].

The prototypic mechanisms of calcium-dependent gene regulation involve the CaMK mediated phosphorylation of enhancers, such as cAMP response element-binding protein (CREB) [20]. In addition, the CREB-independent mechanisms of CaMK-induced gene expression have also been identified [21]. For example, CaMK targets other transcription factors to mediate gene activation, such as NeuroD and LMO4 [22,23] as well as coactivators p300 and CREB-binding protein. The genetic deletion of CaMKIV (but not CREB) in dopamine-D1 responsive neurons selectively potentiated cocaine-induced FosB expression and reinforcement behaviors, suggesting that CaMKIV targets transcription factors other than CREB to regulate gene expression and behavior [24]. We therefore investigated whether CaMKIIα or CaMKIV regulates the transcriptional activity of Freud-1.

We hypothesized that CaMK directly phosphorylates Freud-1 to regulate its function. Here, we address whether CaMKIIα and CaMKIV phosphorylate Freud-1 in vitro and/or in cells, and performed site-directed mutagenesis to elucidate the role of potential CaMK-dependent phosphorylation sites. CaMKIV, but not CaMKIIα, selectively phosphorylated Freud-1 at a threonine site flanking the C2 domain. The transfection of the activated CaMKIV increased the threonine phosphorylation of Freud-1 and inhibited the Freud-1-mediated repression of the 5-HT1A receptor gene, which was blocked by the mutation of the phosphorylation site. The calcium-induced inactivation of Freud-1 provides a novel mechanism by which an increase in the intracellular calcium concentration activates gene expression, by inducing the CaMKIV-dependent phosphorylation of Freud-1 to de-repress the gene expression in neurons.

## 2. Results

### 2.1. Human Freud-1 Phosphorylation by CaMKIIα and CaMKIV In Vitro

**Figure 1:**
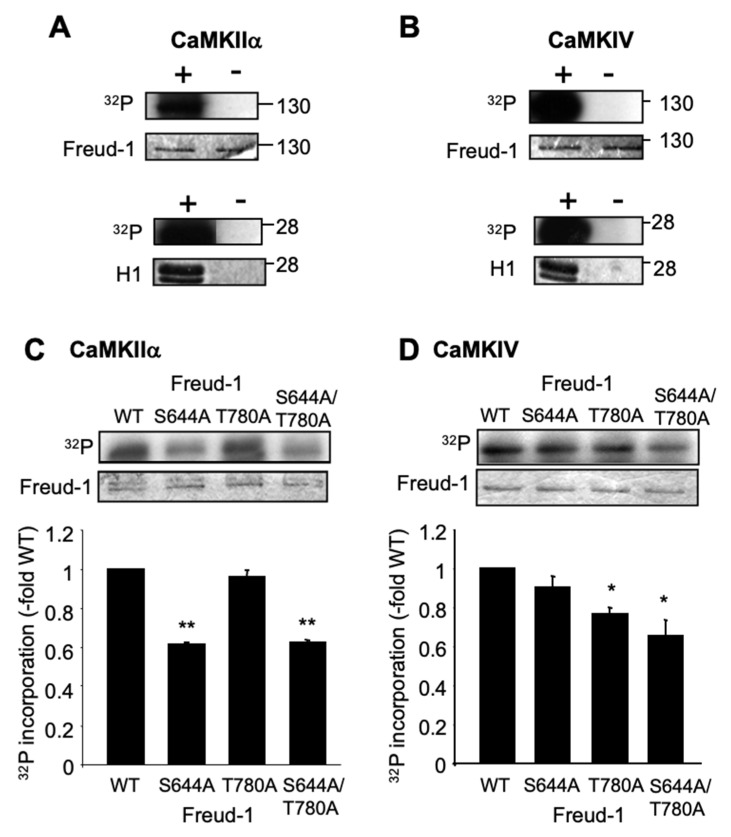
Freud-1 phosphorylation by CaMKIIα and CaMKIV in vitro. (**A**,**B**) In vitro kinase assay. Recombinant purified human Freud-1 (hF1) was incubated in the absence or presence of CaMKIIα (**A**) or CaMKIV (**B**) and [^32^P] incorporation was detected via autoradiography (upper panels); Freud-1 protein was detected in the same gels via Coomassie Blue staining (lower panels). Data are representative of at least three independent experiments. (**C**,**D**) Phosphorylation of Freud-1 mutants. Above: Purified Freud-1 wild-type or mutants Ser644Ala and Thr780Ala or the double mutant were incubated in the presence of CaMKIIα (**C**) or CaMKIV (**D**) and subjected to autoradiography. Purified Freud-1 protein was detected via Western blot using S-tag antibody. Below: Quantification of Freud-1 phosphorylation relative to wild-type (WT) Freud-1. Data represent mean ± SE of three separate experiments; * *p* < 0.05; ** *p* < 0.01 compared to WT, by using one-way ANOVA with Tukey post-test.

We addressed whether purified recombinant human Freud-1 is a direct substrate for CaMKIIα or CaMKIV using in vitro protein kinase assays (Figure 1A,B). In the presence of either activated CaMKIIα or CaMIV, a strong [^32^P]-labeled species corresponding to the full-length human Freud-1 (≈120 kDa) was observed, while in the absence of CaMK, no [^32^P] incorporation was observed. As a positive control, the Histone-H1 phosphorylation by CaMK was also observed. These results provide evidence that Freud-1 is a direct substrate of CaMKIIα and CaMKIV.

To determine the sites of CaMK-induced phosphorylation, the two strongest CaMK consensus phosphorylation sites (Ser644 and Thr780) of human Freud-1 were identified using Scansite (http://scansite.mit.edu accessed on 23 June 2008) [25]. These sites were mutated to non-phosphorylatable Ala residues and tested in vitro for CaMK-mediated phosphorylation (Figure 1C,D). CaMKIIα-induced phosphorylation was reduced by 40% for S644A- and S644A/T780A-Freud-1 compared to wild-type or T780A-Freud-1 (Figure 1C). In contrast, CaMIV-induced phosphorylation was significantly reduced in the T780A and double mutants, but not in the S644A-Freud-1 mutant (Figure 1D). No phosphorylation was observed in the absence of CaMK. The residual phosphorylation in the Freud-1 mutants suggests that CaMK may phosphorylate other consensus sites under these conditions in vitro. These results suggest that Ser644 is a preferred target of CaMKIIα, while Thr780 is a preferred target of CaMKIV for the phosphorylation of Freud-1.

### 2.2. CaMKIV-Mediated Phosphorylation of Freud-1 in Cells

**Figure 2:**
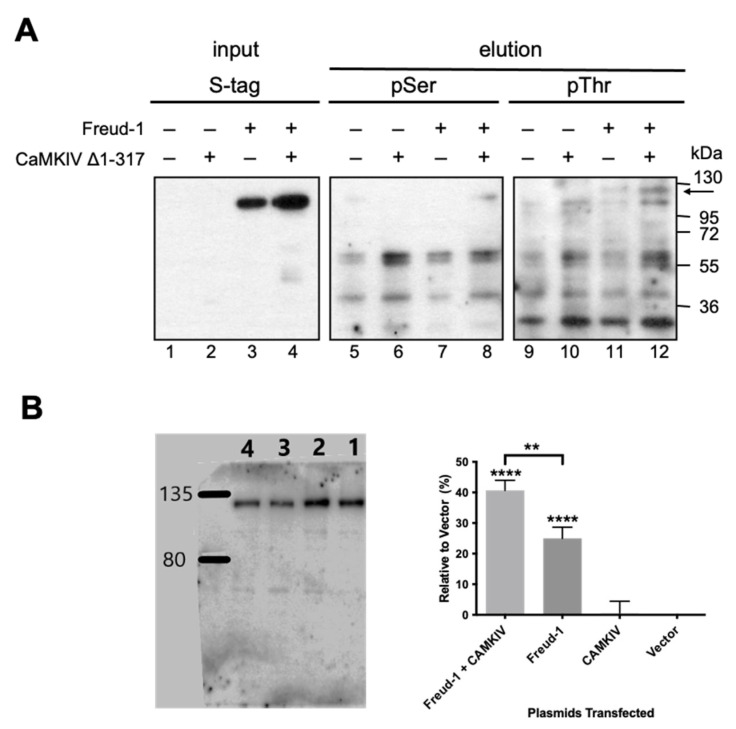
CaMKIV-mediated phosphorylation of Freud-1 in SK-N-SH cells. (**A**) Phospho-serine (pSer)/phospho-threonine (pThr): Lysates from transfected SK-N-SH cells were used in pulldown of His/S-tagged Freud-1. Cells were transfected with plasmids for constitutively active CaMKIV Δ1-317 (+), His/S-tagged Freud-1 (+) or control vector (−). Input and elution fractions were analyzed via Western blot using S-tag antibody for Freud-1 (arrow), and pSer or pThr antibody (1:5000) for phosphorylation. (**B**) Phospho-Thr780: HEK-293 cells were co-transfected with constitutively active CaMKIV Δ1-317 (+) and His/S-tagged Freud-1 (lane 1), vector and Freud-1 (lane 2), CaMKIV (lane 3) or vectors only (lane 4). Western blots were probed with anti-phospho-T780 Freud-1 as shown on a representative blot. Right, band intensities were quantified using Image J win64 Software for densitometry analysis of peak areas. Data are expressed as % above vector control (0%) as mean ± SEM, *n* = 3; **** *p* < 0.0001 vs. vector; ** *p* < 0.01 between groups, one-way ANOVA, Tukey post-test.

To address whether Freud-1 is phosphorylated by CaMK in cells, we transiently transfected CaMKIIα or CaMKIV constructs in human SK-N-SH neuroblastoma cells, a model of 5-HT1A receptor-expressing neurons [26]. CaMKIIα and CaMKIV were weakly detected in non-transfected cells, but equally expressed in cells transfected with wild-type, inactive CaMK (K42M-CaMKIIα [27]; K75E-CaMKIV) or constitutively active CaMK (T286D-CaMKIIα [28], CaMKIV Δ1-317 [29]) (Figure S1A,B). We focused on SK-N-SH cells co-transfected with His/S-tagged wild-type Freud-1 and constitutively active CaMKIV Δ1-317 (Figure 2A). Following transfection, Freud-1 was purified by using Ni-NTA pulldown and Western blot probed with antibodies to S-tag, phospho-serine or phospho-threonine (Figure 2A). The transfected 120-kDa S-tagged Freud-1 was detected using anti-S-tag in both control and CaMKIV-transfected input lanes 3–4, but not vector lanes 1–2. In the pulldown elution from cells co-transfected with CaMKIV Δ1-317 compared to the vector, the phospho-serine antibody recognized 120-kDa Freud-1 (arrow) in the presence but not absence of transfected CaMKIV Δ1-317 and Freud-1 (Figure 2A, lane 8 vs. 7), while without Freud-1, only smaller non-specific species were present (lanes 5–6). The phospho-threonine antibody more strongly detected a specific CaMKIV-sensitive Freud-1 species as a doublet that may represent the pulldown of endogenous Freud-1 with His/S-tagged Freud-1 (Figure 2A, lane 11–12, arrow). As a positive control for the CaMK activity in SK-N-SH cells, transfection with wild-type or constitutively active CaMKIIα or CaMKIV increased the phosphorylation of endogenous CREB, as detected using phospho-Ser or pSer133 antibodies (Figure S1C). These results support the in vitro phosphorylation of Freud-1 by CaMK, and indicate that CaMKIV phosphorylates Freud-1 mainly at threonine residues in neuronal SK-N-SH cells.

In order to test whether CaMKIV specifically phosphorylates Thr780, a phospho-specific antibody to phospho-Thr780-Freud-1 was generated and used for the Western blot analysis of transfected HEK-293 cells (Figure 2B). In vector-transfected cells (lane 4), a single 120-kDa band corresponding to Freud-1 was observed that was significantly increased upon the transfection of Freud-1 (lane 1), but not CaMKIV alone (lane 3). This basal signal may reflect the high basal phosphorylation of Freud-1 in HEK-293 cells rather than non-phosphorylated Freud-1, given the >8-fold specificity of the antibody for the phospho- vs. non-phosphorylated peptide. Upon cotransfection with both CaMKIV Δ1-317 and Freud-1 (lane 2), a significant 50% increase in band intensity was seen compared to Freud-1-transfected cells (lane 1), indicating that CaMKIV increases the Freud-1 phosphorylation of Thr780.

### 2.3. CaMK Activation Decreases the Freud-1 DNA Binding Activity In Vitro

**Figure 3:**
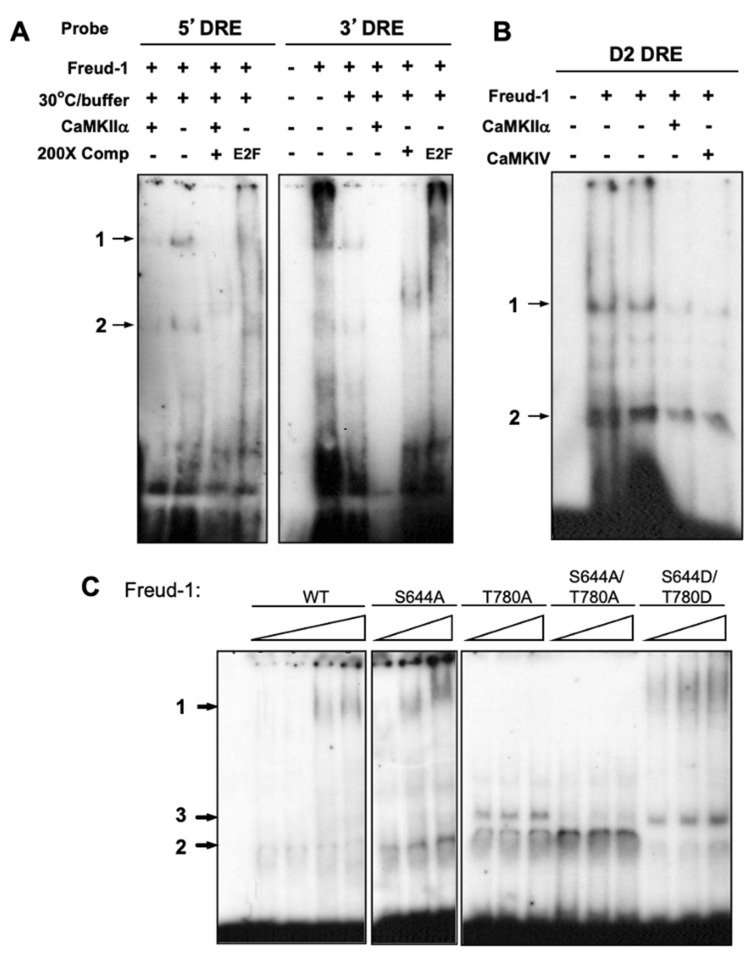
CaMK reduces Freud-1 binding to 5-HT1A and D2 DREs in vitro. Purified Freud-1 was analyzed via EMSA using end-labeled 5-HT1A (5′DRE, 3′DRE) (**A**) or D2-DRE probes (**B**). Two Freud-1/DNA complexes were detected, as observed previously (arrows). Standard conditions or CaMKIIα buffer at 30 °C (30 °C buffer; or all samples in (**B**)) for kinase activity were used, with or without CaMKIIα or CaMKIV, as indicated. Unlabeled 200x molar excess of unlabeled DRE competitor (200XComp) blocked the interaction, but unrelated E2F competitor did not. (**C**) Concentration-dependent Freud-1-DRE complex formation. Bacterially expressed, purified wild-type human Freud-1 (WT) or Freud-1 mutants S644A, T780A, S644A/T780A and S644D/T780D were analyzed via EMSA with labeled 5-HT1A DRE using increasing concentrations (1.5—wild-type only, 2, 2.5, 3 ug) of Freud-1 protein, with no protein added to the first lane. Two complexes were seen, except for T780-containing mutants where a third complex was also seen. Data are representative of at least three independent experiments.

The effect of CaMK on Freud-1 interaction with its DNA elements in the 5-HT1A and dopamine-D2 receptor genes was assessed using in vitro EMSA (Figure 3). Recombinant human Freud-1 was co-incubated with radiolabeled complementary oligonucleotides for the human 5-HT1A 5′-DRE, 5-HT1A 3′-DRE or D2-DRE in the absence or presence of activated CaMKIIα or CaMKIV. As seen previously, in the presence of Freud-1, two major DRE-labeled bands were observed that were competed by unlabeled DRE but not unrelated E2F oligonucleotides [18]. In order to activate CaMK, incubation was performed using CaMK buffer at 30 °C, which slightly reduced the Freud-1-DRE binding in the absence of CaMK (Figure 3A). In the presence of CaMKIIα or CaMKIV, the Freud-1-DRE binding to the 5-HT1A and D2 DREs was almost completely abolished (Figure 3A,B), indicating that the in vitro phosphorylation of Freud-1 by CaMKIIα or CaMKIV reduces its DNA binding activity.

To determine whether the mutation of Freud-1 at sites S644 or T780 affects the binding to DRE complexes, we purified Freud-1 mutants and examined them using an EMSA assay with the 5-HT1A DRE (containing both 5′- and 3′-DRE) (Figure 3C). For wild-type Freud-1, complexes were observed that were not detected in the absence of Freud-1 and increased with increasing Freud-1 protein, and a similar pattern was seen for the S644A mutant. However, Freud-1 mutants containing the T780A mutation displayed a third intermediate migrating species (3) (weaker in the S644A/T780A mutant) with loss of the slow migration complex (1), indicating a change in the Freud-1-DRE binding induced by the T780A mutation. The S644D/T780D mutant, meant to be a phosphomimic, also bound to complexes 1, 2 and 3. However, unlike the other mutants, the S644D/T780D mutant showed reduced binding to phosphatidyl inositol poly-phosphorylated forms [30], suggesting a partial impairment of the Freud-1 function in this mutant. These results indicate that the Freud-1 phospho-site mutants retain binding to the DRE, but that the mutation of the T780 site alters the complex formed.

### 2.4. Activated CaMKIV Attenuates the Freud-1-Induced Repressor Activity

**Figure 4:**
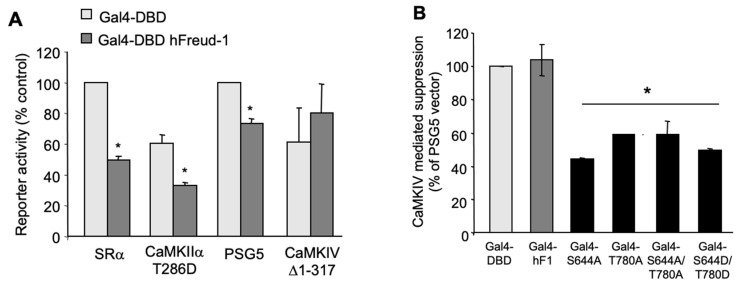
Active CaMKIV but not CaMKIIα attenuates Freud-1 repression. Freud-1 repressor activity was determined using the Gal4 hybrid system (Supplementary Figure S3). (**A**) SK-N-SH cells were co-transfected with reporter constructs G_5_P, Gal4-DBD or Gal4-DBD-Freud-1, and CaMKIIα vector (SRα), constitutively active CaMKIIα T286D, CaMKIV vector (PSG5) or constitutively active CaMKIV Δ1-317. Reporter activity determined in triplicate samples was normalized to β-galactosidase activity and is presented as % of control for each vector. Data represent mean ± SEM of three independent experiments. (**B**) Freud-1 phosphorylation site mutants are resistant to CaMKIV suppression. SK-N-SH cells were transfected with G_5_P, CaMKIV vector (PSG5) or CaMKIV Δ1-317 and Gal4 DBD, Gal4 DBD hFreud-1 or Gal4-DBD Freud-1 mutants. Values for reporter activity are expressed as %CaMKIV-induced suppression = 100 × [CaMKIV Δ1-317]/[PSG5] of reporter activity for each Gal4-Freud-1 construct normalized to the ratio for Gal4-DBD vector (100%). Triplicate transfections from three independent experiments are presented as mean ± SEM. * *p* < 0.05; via unpaired two-tailed *t*-test.

In order to address the action of CaMK on Freud-1-mediated repression, we used the Gal4-hybrid system, which allows the assessment of the repressor activity independent of DNA binding. This system uses the Gal4 DNA binding domain (Gal4-DBD) to recruit Freud-1 to DNA and induce gene repression [31]. Constructs consisting of the yeast, Gal4-DBD, alone or fused to human Freud-1 (Gal4-DBD hFreud-1) were co-transfected in SK-N-SH cells with a luciferase reporter construct containing five (G_5_P) copies of the Gal4 DNA element (Figure S2A). Compared to the Gal4-DBD vector, Gal4-DBD hFreud-1 significantly reduced the reporter activity by almost 50% (Figure S2B,C). These constructs were then co-transfected with activated CaMK constructs or their vectors (Figure 4). Significant Freud-1 induced repression was observed in both the vector (SRα) and active CaMKIIα T286D transfected cells, but Freud-1 repression was abolished upon the transfection of the active CaMKIV Δ1-317 compared to the PSG5 vector (Figure 4A). Thus, while the active CaMKIIα did not affect the Freud-1-mediated repression, the activated CaMKIV blocked the Freud-1 repressor activity.

Since CaMKIV induced the serine and threonine phosphorylation of Freud-1, the importance of Freud-1 phosphorylation sites, Ser644 or Thr780, for CaMKIV to inactivate the Freud-1 repressor function was addressed. The Gal4 reporter construct, G_5_P, was co-transfected in SK-N-SH cells with either vector (Gal4-DBD), wild-type or phospho-site mutant Freud-1-Gal4-DBD in the absence (PSG5 vector) or presence of active CaMKIV Δ1-317 and the % suppression of the Freud-1 repressor activity was calculated (Figure 4B). In the presence of wild-type CaMKIV, Gal4-DBD hFreud-1 displayed no significant repression (see Figure 4A), corresponding to 100% suppression. Conversely, each of the Freud-1 mutant constructs (S644A, T780A, S644A/T780A) repressed the transcriptional activity in the presence of constitutively active CaMKIV Δ1-317. Similarly, the Freud-1 S644D/T780D mutant also repressed the transcriptional activity in the presence of CaMKIV. Thus, the Freud-1 either Ser644 or Thr780 site is required for CaMKIV-mediated inhibition of the Freud-1 repressor activity.

### 2.5. CaMKIV Inactivates the Freud-1-Induced Repression of the Human 5-HT1A Promoter

The effect of the activation of endogenous CaMK by increasing the intracellular calcium on Freud-1-mediated repression at the 5-HT1A DRE was examined in SK-N-SH cells. We tested whether the 5-HT1A DRE elements can confer calcium-dependent regulation on a heterologous (SV40) promoter. Cells transfected with the pGL3P vector or pGL3P 5′ and 3′ DRE containing both DREs upstream of the SV40 promoter were compared (Figure S3A). The presence of the DREs conferred a 30% reduction in the SV40 promoter activity compared to pGL3P, due in part to endogenous Freud-1. Upon treatment with calcium mobilizing agents (depolarizing KCl and calcium ionophore ionomycin), the DRE-mediated repression of the SV40 activity relative to the vector was lost, suggesting that calcium signaling prevents Freud-1-mediated repression in SK-N-SH cells.

**Figure 5:**
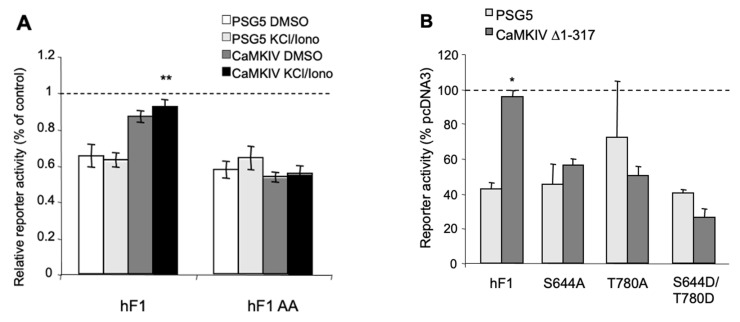
Repression by Freud-1 phospho-site mutants is resistant to active CaMKIV. (**A**) Calcium-CaMKIV-mediated de-repression of 5-HT1A-DRE. SK-N-SH cells were co-transfected with 5-HT1A DRE-SV40 promoter-luciferase construct, CaMKIV or vector (PSG5) and human Freud-1 (hF1) or S644A/T780A-Freud-1 (hF1 AA) or vector (pcDNA3). Cells were treated with vehicle (DMSO) or 40 mM KCl and 1 uM ionomycin for 4 h prior to cell collection. Relative luciferase activity from triplicate samples was normalized to activity in pcDNA3 vector samples (dashed line). Mean ± SEM (*n* = six experiments). ** *p* < 0.01 vs. DMSO control, ANOVA with Bonferroni post-test. (**B**) Freud-1 repression of the 5-HT1A promoter. SK-N-SH cells were co-transfected with human 5-HT1A promoter-luciferase constructs, -1517 (lacking DREs) or -1790ΔRE-1 containing the 5′ and 3′-DRE with the RE-1 deleted [31] and Freud-1 expressing plasmid or vector pcDNA3. Samples were assayed for reporter activity and are presented as % of activity of the DRE-lacking -1517 construct (see Supplementary Figure S3). * *p* < 0.05; ** *p* < 0.01 by unpaired two-tailed *t*-test.

The role of CaMKIV in calcium signaling to regulate Freud-1-mediated repression was examined by triggering a calcium influx using depolarizing KCl and ionomycin treatment in cells transfected with CaMKIV (Figure 5A). SK-N-SH cells were co-transfected with the pGL3P 5′ and 3′ DRE construct, Freud-1 expression plasmids or the vector (control), and wild-type CaMKIV or the PSG5 vector (Figure 5A). The transfection of Freud-1 or S644A/T780A-Freud-1 conferred a significant 35–40% repression compared to the control, which was not affected by the KCl/ionomycin treatment. The lack of a response to the calcium mobilization may be due to the over-expression of Freud-1 in these cells. The transfection of wild-type CaMKIV abolished Freud-1-induced repression, in the absence or presence of calcium mobilization, suggesting that over-expressed CaMKIV is active under basal calcium conditions. However, in cells transfected with S644A/T780A-Freud-1, Freud-1-induced repression was resistant to CaMKIV, without or with calcium mobilization, suggesting a key role for these sites in the CaMKIV-induced inactivation of Freud-1.

The role of CaMKIV to regulate the Freud-1-mediated repression of the full human 5-HT1A promoter construct in SK-N-SH cells was determined. For optimal promoter activity, we used the −1790ΔRE-1 5-HT1A luciferase reporter construct which contains the 5-HT1A promoter and 5′/3′-DREs and is repressed by Freud-1, but lacks the RE-1 element [31] (Figure S3B). As a negative control, Freud-1-induced repression was blocked in the -1517 5-HT1A promoter construct, in which the DRE and RE-1 elements are deleted (Figure S3B). The transfection of the 1790ΔRE-1 promoter construct with Freud-1 resulted in a 60% repression compared to the Freud-1 vector control (pcDNA3), and this repression was completely reversed by the co-transfection of active CaMKIV Δ1-317 (Figure 5B). By contrast, the S644A- and T780A-Freud-1 mutants retained full repressor activity at the 5-HT1A promoter upon the co-transfection with active CaMKIV. The S644D/T780D double mutant also displayed repressor activity that was insensitive to CaMKIV and may reflect the less acidic pKa of aspartic acid compared to the phosphorylation sites. The repressor function of these mutants is consistent with their binding to the 5-HT1A DRE (Figure 3C). Together, these results show that Freud-1 phosphorylation sites Ser644 and Thr780 are necessary for CaMKIV to block the Freud-1-mediated repression of the 5-HT1A receptor gene.

## 3. Discussion

### 3.1. CaMKIV Inhibits the Freud-1 Repression of the 5-HT1A Receptor Gene

Previously, we found that calcium mobilization induces 5-HT1A promoter activity, an action dependent on 5-HT1A-DREs and reversed by CaMK inhibitor KN93, suggesting a role for the CaMK-induced inhibition of Freud-1 repressor activity [17]. Here, we examined the role of CaMK in the calcium-dependent regulation of human Freud-1. We found that both CaMKIIα and CaMKIV phosphorylated human Freud-1 in vitro at specific sites and show that in cells, only CaMKIV phosphorylates Freud-1 at serine and threonine residues (including threonine 780) to inhibit its repressor activity. Compared to CaMKIIα, CaMKIV is located predominantly in the nucleus and is known to influence the transcription factors related to long-term memory [32,33,34,35]. Freud-1 is mainly localized in the nucleus in SK-N-SH cells and neurons, but is also present in the cytosol [18]. The nuclear localization of CaMKIV compared to cytosolic CaMKIIα may account for its preferential role in regulating the Freud-1 repressor function in cells. In nuclear extracts, the addition of Ca/ATP was sufficient to reduce the Freud-1-DRE binding [17], consistent with the activity of the endogenous CaMKIV and Freud-1 in the nucleus. Using three different transcriptional reporter systems, we also found a primary role of CaMKIV in the calcium-mediated attenuation of the Freud-1-mediated repression in cells, consistent with its role in transcriptional regulation [34]. By contrast, constitutively active CaMKIIα showed no effect on the Freud-1 repressor activity in the Gal4 system. Thus, our data indicate that CaMKIV is the predominant mediator of the calcium-dependent inactivation of Freud-1 in SK-N-SH cells.

In contrast to the inactivation of Freud-1, CaMKIV has been shown to mediate the calcium-induced phosphorylation and activation of several transcriptional enhancers, such as CREB and LMO4 [23,34] and of co-activators such as the CREB binding protein (CBP) and p300 [32,36]. The CaMKIV-mediated inactivation of transcriptional repressors like Freud-1 has not been previously reported, although the CaMKIV-induced phosphorylation and translocation of histone deacetylase (HDAC) 4 to the cytoplasm has been shown to enhance MEF2 activity [37,38]. Thus, the calcium-mediated inactivation of repressor activity could be synergistic with the CaMKIV-mediated activation of transcriptional enhancers [20]. 

### 3.2. Sites of the CaMKIV-Induced Phosphorylation and Inhibition of Freud-1

**Figure 6:**
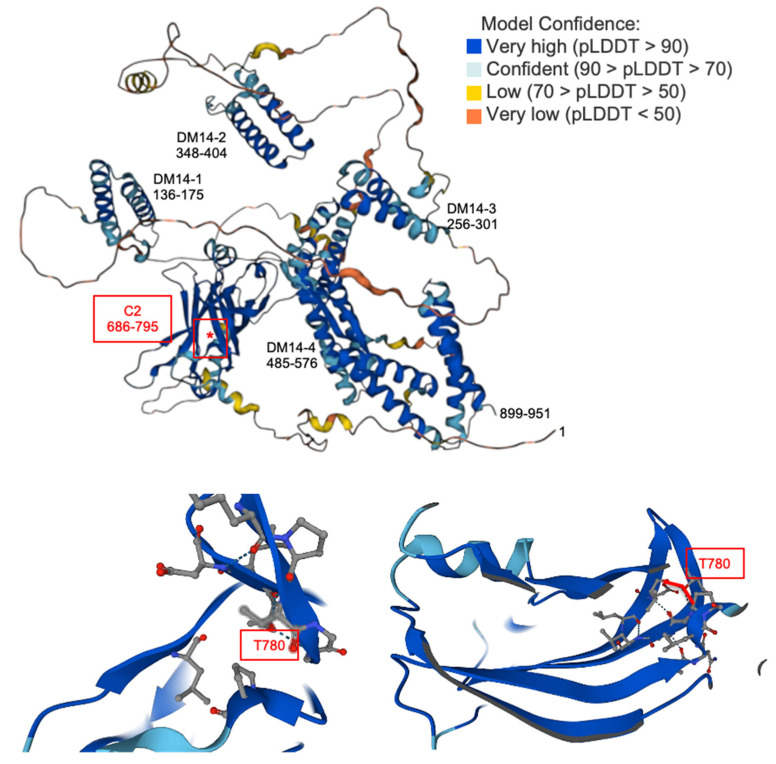
Structural model of Freud-1 domains. Freud-1/CC2D1A 3D structure predicted by AlphaFold [44] was obtained at: https://www.genecards.org/cgi-bin/carddisp.pl?gene=CC2D1A (accessed on 11 March 2024). Shown are the Freud-1 DM14 and C2 domains as predicted by the model and the site of the T780 residue (red asterisk). Below are blow-ups of the boxed region in two different rotations which show hydrogen bonding of the T840 (in red): T780-OH forms coordinate hydrogen bonds with V773=O…HO–T780 and G782–N…T780–OH.

Two consensus CaMK phosphorylation sites were identified in human Freud-1, Ser644 (^548^FEQRTFSVIKI^558^) and Thr780 (^773^VLDGRRPTGGRL^784^) (underlined), but only the Thr780 site is conserved in mouse Freud-1. Although the CaMKIV sites that we identified have negatively charged residues at the P-5 site (bold) whereas hydrophobic residues are predicted from peptide studies [39], studies of prohibitin2, CBP and p300 show that this consensus sequence may not be as stringent in the context of the tertiary protein structure [32,36]. In the case of Freud-1, the adjacent hydrophobic residues at P-6 and P-4 may substitute for the P-5 site. The residual CaMKIIα-mediated phosphorylation of the S644A/T780A Freud-1 mutant in vitro may represent the phosphorylation at atypical consensus sites, such as an S/TXD phosphorylation site (Ser220) in human Freud-1 that is not conserved in mouse or rat Freud-1 [40]. Although excess CaMKIIα may phosphorylate atypical sites in vitro, CaMKIIα-induced serine phosphorylation was not observed in cells. By contrast, CaMKIV increased the Freud-1 phosphorylation at serine and predominantly threonine residues, both in vitro and in cells. In addition to Ser220, Ser208 has been shown to be phosphorylated by CDK1 [41], and CaMKIV may indirectly activate CDK1 to phosphorylate this site. The CaMKIV-induced phosphorylation of Thr780 in cells suggests that this may be a key site for CaMKIV action. Thr780 is conserved in Freud-2/CC2D1B, which also binds the 5-HT1A-DRE [42,43], suggesting that it too may be inhibited by CaMKIV activation. The Thr780 site lies in the last beta-sheet of the conserved C2 domain of Freud-1 and forms coordinate hydrogen bonds with V773 and G782 to form a loop in the predicted model (Figure 6). Thus, the phosphorylation of Thr780 may disrupt these bonds by steric hindrance or ionic bonding to prevent Freud-1 DNA binding and repressor functions.

Previous evidence indicates that the C2 domain is critical for Freud-1-DNA binding and repressor function. In particular, an 8-amino acid deletion at the N-terminal side of the C2 domain (amino acids 696–703) attenuated Freud-1 binding to the DRE and completely blocked Freud-1 repressor activity [17]. Consistent with its role in DNA binding, we found that the mutation of the Thr780 site altered the Freud-1-DNA complexes formed in vitro. However, the mutation of the S644 or T780 sites to alanine or aspartate did not prevent Freud-1-DRE binding in vitro or the Freud-1-mediated repression in cells, indicating that these phosphorylation-resistant mutants remain functional. By contrast, unlike the alanine mutants, the S644D/T780D did show reduced binding to phosphatidyl inositol poly-phosphates, suggesting a partial impairment of this mutant [30]. Importantly, these mutants retained repressor function but prevented the inactivation of Freud-1 by CaMKIV, indicating the role of Ser644 and Thr780 phosphorylation in the CaMKIV-induced inactivation of Freud-1 repressor activity.

### 3.3. Freud-1-Containing DNA Complexes

We have previously characterized the 5-HT1A DRE as containing two homologous elements, 5′- and 3′- repressor elements FRE and TRE, respectively [17]. The FRE preferentially recognizes Freud-1, while the TRE binds Freud-2/CC2D1B [43]. In EMSA assays, we found two protein complexes of purified wild-type human Freud-1 with 5-HT1A 5′- and 3′-DREs and the D2 DRE. Since each DRE contains two Freud-1-like elements, the low mobility complex may represent two molecules of Freud-1 bound to the DRE, while the higher mobility complex may contain only one molecule bound. Interestingly, a third complex formed with the Freud-1 Thr780 mutants, suggesting that the point mutation may alter the Freud-1 structure to affect its migration. Nevertheless, these Freud-1 mutants retained the function to repress 5-HT1A receptor expression.

As a repressor, Freud-1 recruits several different transcriptional regulators including the SWI/SNF chromatin remodeling complex, Brg1-BAF170/57, and corepressor complex, Sin3A-HDAC1/2, the latter being absent in SK-N-SH cells compared to HEK-293 cells [45]. Thus, the Freud-1-mediated repression was blocked by the HDAC inhibitor, trichostatin, in HEK-293 cells but not in SK-N-SH cells, indicating HDAC-independent repression [31]. The ability of Freud-1 S644 and T780 mutants to bind and repress at the 5-HT1A DRE suggests that these mutations do not impair the recruitment of key regulators, but it is possible that the mutations may shift the HDAC dependence of the Freud-1 repressor function. In addition to interacting with transcriptional regulators, Freud-1 also interacts with a variety of phosphatidyl inositol 3′-phosphate lipids [30] and cytoplasmic proteins including PDK1-Akt scaffolding, endocytotic CHMP-4B/4C and centrosome cohesin proteins [3]. However, since nuclear-localized CaMKIV blocked the Freud-1 repressor function while CaMKIIα did not, this indicates that nuclear rather than cytoplasmic phosphorylation may be critical.

### 3.4. Potential Biological Roles for the CaMKIV-Dependent Inactivation of Freud-1

In addition to Freud-1, several transcriptional and chromatin regulatory factors have been linked with intellectual disability [46]. Interestingly, Freud-1 is expressed widely throughout the brain and during development and has been indirectly implicated in cortical development [4,17]. A deletion in the Freud-1 gene (CC2D1A) that removes the critical C2 domain has been linked to non-syndromic intellectual disability in several cohorts [4,5]. Mice with a germline deletion of Freud-1 die after birth and show an impaired differentiation of the cortical neurons [47]. Conditional knockout of Freud-1 in the early post-natal forebrain results in impaired learning and memory and increased anxiety [5,7]. Thus, the CaMKIV-induced inactivation of Freud-1 may play a role in normal cognitive and emotional development in part through the induction of downstream Freud-1 target genes, including the 5-HT1A and dopamine-D2 receptor genes.

As a key regulator of 5-HT1A receptors, Freud-1 has also been implicated in depression. Freud-1 is co-expressed with 5-HT1A receptors widely throughout the brain, especially in the hippocampus, cortex and in raphe serotonergic neurons where it regulates 5-HT1A autoreceptors [17,48,49]. In subjects with depression compared to controls, the levels of Freud-1 in the prefrontal cortex were reduced, particularly in male subjects under 50 years old, and 5-HT1A receptors were also down-regulated [48]. Consistent with its repressor function, in chronic restraint stress depression models in male rats and mice, Freud-1 RNA was reduced, while 5-HT1A RNA was increased in the cortex and raphe, respectively [50,51]. However, the stress-induced reduction of the Freud-1 protein levels sometimes correlated with reduced 5-HT1A receptor expression suggesting that post-transcriptional Freud-1 or 5-HT1A regulation may be more important [52].

Alterations in Freud-1 phosphorylation may also be implicated in antidepressant action. The firing of 5-HT neurons is thought to release 5-HT in the raphe nuclei which reduces 5-HT firing through negative feedback via the inhibitory signaling of 5-HT1A autoreceptors, which is increased by antidepressants that inhibit 5-HT reuptake [53,54]. However, chronic treatment with 5-HT1A receptor agonists or 5-HT reuptake inhibitors leads to the gradual down-regulation of 5-HT1A autoreceptors [55,56,57] and RNA levels [58]. The gradual desensitization and down-regulation of 5-HT1A autoreceptors following chronic antidepressant treatment is seen clinically and may account for the 3-week latency of the clinical effectiveness of these compounds [59]. Oppositely, the conditional knockout of Freud-1 in 5-HT neurons resulted in the over-expression of 5-HT1A autoreceptors and the reduced activity of 5-HT neurons and led to anxiety and depression-like phenotypes that were resistant to chronic fluoxetine treatment [10]. Importantly, the effect of the actions of Freud-1 knockout in serotonin neurons on behavior required the presence of 5-HT1A autoreceptors, indicating that their up-regulation prevents the fluoxetine response. Consistent with this, depressed subjects [60] and subjects at a high familial risk of depression [61] have higher levels of 5-HT1A autoreceptors, and are more likely to be diagnosed with depression and to be non-remitters [62]. Our current results indicate that the calcium-dependent induction of 5-HT1A receptor expression could be mediated by CaMKIV-Freud-1 signaling. The glutamatergic projection from the prefrontal cortex to the raphe activates 5-HT neurons [63], inducing antidepressant effects [64]. As a negative feedback pathway, the calcium/CaMKIV-mediated inactivation of Freud-1 to induce 5-HT1A autoreceptor levels would result in feedback to inhibit serotonin neuron firing [10]. Similarly, excessive 5-HT1A autoreceptor signaling (e.g., following acute SSRI treatment) would reduce calcium levels [65], releasing the CaMKIV-mediated inhibition of Freud-1 to repress 5-HT1A gene transcription [54]. Thus, the CaMKIV-induced inactivation of Freud-1 could provide an important mechanism for the activity-dependent regulation of gene transcription implicated in anxiety, depression and the response to antidepressants.

Finally, the CaMKIV-induced inactivation of Freud-1 could be particularly important in regions such as the prefrontal cortex or hippocampus, which are enriched in Freud-1 and CaMKIV and are where CaMKIV mediates the long-term changes in gene expression, synaptic development and cognitive function [20]. In support of this, the inactivation of Freud-1 in the early post-natal forebrain via gene deletion in mice leads to impairments in cognitive function [5,7], consistent with the mutations in Freud-1 causing intellectual disability in humans.

## 4. Materials and Methods

### 4.1. Plasmid Construction and Mutagenesis

The human long Freud-1 [1] subcloned into pcDNA3 (Invitrogen, Burlington, ON, Canada) and pTriEx4 (Novagen, Madison, WI, USA) [18] was mutated using the QuickChange^®^ II XL Site Directed Mutagenesis Kit (Stratagene, La Jolla, CA, USA) with appropriate mutagenic primers. The DRE-pGL3P and 5-HT1A promoter-pGL3B luciferase constructs, and Gal4-Freud-1 fusion constructs were as described in [31]. Rat αCaMKII, K42M, human CaMKIV, K75E and Δ1-317 constructs were kindly provided by Dr. A.R. Means (Duke University, Durham, NC, USA), originally from Dr. H Schulman (Stanford University, Stanford, CA, USA) and SRα [66] or pSG5 vectors (Stratagene) were obtained via the excision of inserts and re-ligation. The constitutively active T286D CaMKIIα mutant was generated via site-directed mutagenesis. All plasmids were verified via automated sequencing and quantified spectrophometrically and via ethidium bromide staining.

### 4.2. Cell Culture and Transient Transfection

Human HEK 293 cells and SK-N-SH neuroblastoma cells were maintained in DMEM (Wisent, St-Bruno, QC, Canada) with 10% FCS/1% penicillin/streptomycin at 37 °C in 5% CO_2_. Cells grown to 50–60% confluence were transiently transfected via calcium phosphate co-precipitation using 0.5 μg of the reporter construct, 0.5 μg of human Freud-1 expression plasmid or the vector, 0.5 μg of CaMK or the vector and 0.1 μg/well of pCMVβgal (Clontech, Mountain View, CA, USA). For protein expression, 5 μg of plasmid/10 cm plate was used; for the pulldown assay or immunoprecipitation, 3 μg of plasmid/10 cm plate was used.

### 4.3. Freud-1 Protein Expression and Purification

E. coli BL21 (DE3) (Novagen) was transformed with pTriEX4- hFreud-1 plasmid, grown overnight and induced at OD600 = 0.6 with 1 mM isopropyl-β-D-thiogalactopyranoside (IPTG; Wisent) at 37 °C for 3 h. Cells were lysed, and supernatant proteins were purified on a Ni-NTA matrix column (Qiagen) as previously described in [18]. Fractions containing human Freud-1 were dialyzed for 16 h in PBS or DNA-binding buffer containing 20 mM HEPES pH 7.9, 0.2 mM EDTA, 0.2 mM EGTA, 100 mM KCl, 5 mM MgCl_2_, 5% glycerol and 2 mM dithiothreitol and stored at −80 °C.

### 4.4. CaMK Kinase Assay

Purified CaMKIIα (500 U/μL) or CaMKIV (500 U/μL) (New England Biolabs Inc. Pickering, ON, Canada) was pre-activated at 30 °C for 10 min in CaMK buffer with 2 mM CaCl_2_, 1.2 μM calmodulin and 100 μM ATP, then incubated with 1 μg of purified His/S-tag-Freud-1 (see Section 4.3) or purchased Histone-H1 (Sigma-Aldrich, Saint Louis, MO, USA) was placed in CaMK buffer and 1 μCi [γ-^32^P] for 30 min at 30 °C. The samples were resolved by using 10% SDS-PAGE, stained with Coomassie Blue, dried and ^32^P incorporation visualized via autoradiography.

### 4.5. Pulldown Assay

Cells were washed with PBS, collected via centrifugation, re-suspended in lysis buffer with protease inhibitors (Roche, Laval, QC, Canada) and incubated for 30 min at 4 °C, and then sonicated and centrifuged at 1000× *g* 5 min to remove cell debris. Cleared lysate was loaded on Ni-NTA Spin Columns (Qiagen, Mississauga, ON, Canada), centrifuged for 2 min at 1000× *g*, washed 3× and eluted 3× with 200 μL of elution buffer. Lysates and purified proteins were quantified using the BCA Protein Assay Kit (Pierce Biotechnology, Rockford, IL, USA) and subjected to Western blot analysis.

### 4.6. Immunoprecipitation (CREB)

After transfection (48 h), cells washed in PBS were lysed with RIPA buffer for 2 h at 4 °C, and then sonicated and centrifuged for 10 min at 1000× *g*. The supernatant was precleared in 60 μL of Protein A4 Fast Flow (GE HealthCare, Baie d’Urfe, QC, Canada) for 30 min at 4 °C and CREB antibody (1:250) was added; the samples were incubated for 16 h at 4 °C. Protein A beads were added, incubated for 2 h at 4 °C, washed 5× with lysis buffer, eluted in 60 μL of SDS loading buffer and analyzed by using Western blot.

### 4.7. Phospho-T780-Freud-1 Antibody

A phospho-specific rabbit polyclonal antibody for the T780 CaMK phosphorylation site of human Freud-1 (NP_060191) was generated using the peptide sequence at amino acids 775-785: C-DGRRP(pT)GGRLE. An N-terminal cysteine was included that was conjugated to Keyhole Limpet Hemocyanin as the hapten for antibody generation (Abmart Inc., Shanghai, China). The antibody was antigen affinity-purified at a phospho/unmodified titer ratio ≥ 8 via ELISA. HEK-293 cells seeded at 4 × 10^5^ cells/6-well plate were transfected with 0.5 µg each of S-tagged human Freud-1 or vector pTriex4 and delta1-314 CaMKIV or vector pSG5, and with 0.5 µg of pGL3P and 0.1 µg of pCMV beta-gal to measure the transfection efficiency which was equal in all transfections. Cells were harvested in RIPA buffer and stored at −80 °C. For Western blot, 20 µg/lane of total protein was run on 6% polyacrylamide gel; 1:5000 Anti-pT380-Freud-1 was used. The protein ladder was from New England Biolabs (P7712S).

### 4.8. Electrophoretic Shift Mobility Assay (EMSA)

Gel-purified complementary oligonucleotides with GG overhangs (Table 1) were denatured at 100 °C for 5 min, annealed and labeled with [α^32^P] dCTP using 2.5 U of Klenow (New England Biolabs) and purified on a Sephadex G-50 column (GE HealthCare). Purified protein was pre-incubated with or without annealed unlabeled oligonucleotides (Table 1) in a 25 μL reaction containing DNA binding buffer and 250 ng of herring sperm DNA (Roche) at 22 °C for 30 min. A [^32^P]-labeled probe (50,000 cpm/sample) was added and incubated for 30 min at 22 °C. The reaction was separated on a 4% non-denaturing polyacrylamide gel at 4 °C, dried and visualized via autoradiography. Purified protein was treated with activated CaMK at 30 °C for 10 min before the DNA binding reaction.

### 4.9. Western Blot Analysis

Following transfection (48 h), cells were washed with PBS, collected by centrifugation, and re-suspended in RadioImmuno Precipitation Assay (RIPA) buffer (50 mM Tris, 150 mM NaCl, 0.1% *w*/*v* SDS, 0.5M EDTA, 1% *w*/*v* NP-40, 0.5% *w*/*v* deoxycholate sodium salt, 10 mM sodium fluoride, 10 mM disodium pyrophosphate) with 1x Complete protease inhibitor (Roche) and incubated for 30 min at 4 °C, sonicated and protein quantified using the BCA Protein Assay (Pierce). SDS loading buffer was added, samples were boiled for 5 min, separated using SDS-PAGE and transferred to a polyvinylidene fluoride (PVDF) membrane (NEN, Waltham MA) and washed at 4 °C in 2% blocking solution (Roche) in Tris buffered saline (TBS) (50 mM Tris, 150 mM NaCl, pH 8.0). The blots were incubated for 1 h with 1:5000 HRP-conjugated S-tag antibody (Novagen) in blocking solution, washed 3 × 10 min at 22 °C in TBS-T (TBS, 0.1% Tween 20) and developed using BM Chemiluminescence Blotting Substrate (Roche). Antibodies to CREB and phospho-CREB Ser133 (1:5000 overnight, Cell Signaling Technologies Inc. Danvers, MA, USA), phosphoserine or phosphothreonine (1:5000 overnight, Sigma-Aldrich), CaMKIIα (1:5000 overnight, Santa Cruz Biotechnology Inc. Santa Cruz, CA, USA #9035), CaMKIV (1:5000 overnight, Cell Signaling Technologies Inc., #4032) and β-actin (1:5000 for 2 h, Sigma-Aldrich) were also used. Membranes were washed (2 × 10 min, 22 °C in blocking solution; 1x 10 min TBS-T) and secondary antibodies were added, including horseradish peroxidase-conjugated anti-rabbit (1:5000, 2 h, Cell Signaling Technologies Inc.) and anti-mouse (1:5000, 2 h, Jackson Laboratories Bar Harbor, MA, USA).

### 4.10. Luciferase and β-Galactosidase Assays

After transfection (48 h), triplicate wells were washed with PBS, 100 μL/well of reporter lysis buffer (Promega) was added, freeze-thawed, centrifuged (10 min, 1000× *g*, 4 °C) and 30 μL of supernatant was added to 100 μL of luciferase buffer and assayed for luciferase activity using LMax II 348 (Molecular Devices, Sunnyvale, CA, USA). The β-galactosidase activity was assayed by adding 30 μL of the sample to 200 μL of β-galactosidase substrate buffer (1xPBS, 0.5 mM MgSO_4_, 1.35 mL of β-mercaptoethanol) and 10% of 4 mg/mL chlorophenolred-β-D-galactopyranoside (CPRG; Calbiochem, San Diego, CA, USA). To correct for transfection efficiency, luciferase values were normalized to β-galactosidase activity.

### 4.11. Statistical Analysis

Data are presented as mean ± SEM. ANOVA was used for multiple comparisons, and Student’s *t*-test (2 tailed, unpaired equal variance) was used when two groups were compared; significance is represented as * *p* < 0.05, ** *p* < 0.01.

## 5. Conclusions

We provide evidence that CaMKIV mediates the phosphorylation of Freud-1 at serine and threonine sites, particularly Thr780. Calcium-CaMKIV-induced phosphorylation prevents Freud-1 binding to 5-HT1A-DRE and D2-DRE sites and blocks Freud-1-mediated repression of the 5-HT1A promoter. The calcium-induced inactivation of Freud-1 via CaMKIV activation and the resulting induction of 5-HT1A receptor expression may provide an important mechanism implicating cognitive and behavior development and function, as well as in response to chronic antidepressant treatment.

## Figures and Tables

**Table 1:** Sequence of oligonucleotides for 5-HT1A and D2DR receptor DNA elements.

Name	Sequence
5′-DRE	5′-AGATGGCACTCTAAAACATTTGCCACA
3′-DRE	5′-AGGTGGCGACATAAAACCTCATTGCTTAGAACT
5′+3′-DRE	5′-AGATGGCACTCTAAAACATTTGCCACAAGGTGGCGACATAAAACCTCATTGCTTAGAACT
hD2DR DRE	5′-CGCGTGGGATAAGCAAGCCCTTCTTGTAAAAGTTTTAAGAACAATACA
E2F	5′-TATAGTGTACTCTACTATTCTGCTC

Shown are the sequences for the dual repressor elements (DREs) of the human 5-HT1A receptor gene, human D2DR gene and the E2F element, used as a non-specific competitor. These sense and complementary antisense oligonucleotides were hybridized and used for electrophoretic mobility shift assays (EMSA) and for generating reporter constructs.

## Data Availability

Data are available upon request to corresponding author.

## Supplementary Materials

The following supporting information can be downloaded at: https://www.mdpi.com/article/10.3390/ijms25116194/s1.
