# Supplementary material for: CaMKIV-Mediated Phosphorylation Inactivates Freud-1/CC2D1A Repression for Calcium-Dependent 5-HT1A Receptor Gene Induction
Source: Int J Mol Sci. 2024 Jun 4;25(11):6194. doi: 10.3390/ijms25116194 (PMC11172825; doi:10.3390/ijms25116194)
Supplement: Supplementary file 1 [file ijms-25-06194-s001.zip › ijms-3008598-supplementary.pdf]

Figure S1

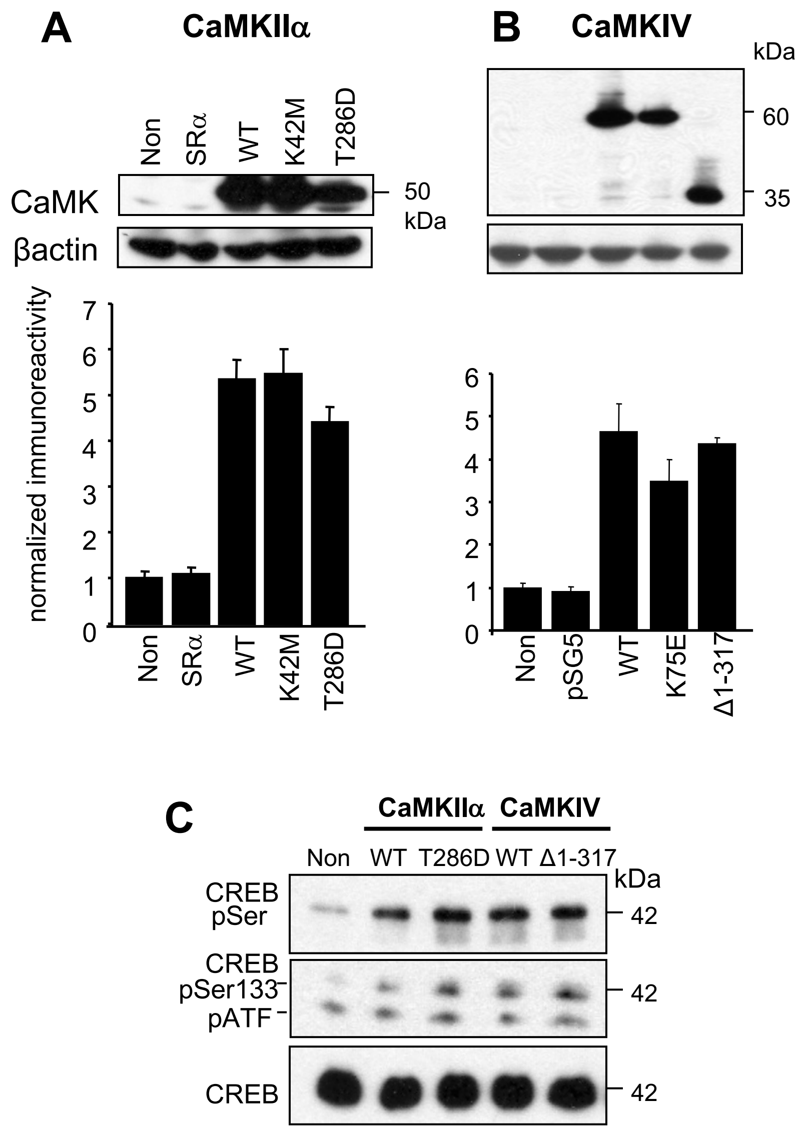

Figure S1: Transfection of CaMKIIa and CaMKIV and CREB phosphorylation.

A-B: Transfection of CaMK. SK-N-SH human neuroblastoma cells were transiently transfected or not (Non) with the indicated plasmids: A. CaMKIIα: Vector (SRα); wild-type (WT); kinase-deficient (K42M); or constitutively active (T286D) CAMKIIα. B. CaMKIV: Vector (pSG5); wild-type (WT); kinase-deficient (K75E); or constitutively active (D1-317) CaMKIV. CAMK protein was detected by western blot using anti-CaMKIIα (A) or anti-CaMKIV (B); β-actin was detected as a loading control. Below: Protein expression was quantified and normalized to non-transfected control (Non) and is shown as Mean ± SE (n=3). C: CaMK-mediated CREB phosphorylation in SK-N-SH cells. Lysates from SK-N-SH cells not transfected (Non) or transiently transfected with constructs for CaMKIIα wild-type (WT) or active mutant (T286D); or CaMKIV wild-type (WT) or active mutant (Δ1-317) were immunoprecipitated using anti-CREB antibody and subjected to Western blot analysis. Anti-phospho-serine was used to detect CREB serine phosphorylation (pSer); anti-phospho-CREB antibody for CREB Ser133 phosphorylation (pSer133) as well as ATF phosphorylation (p-ATF); or anti-CREB as loading control to detect total CREB.

Figure S2

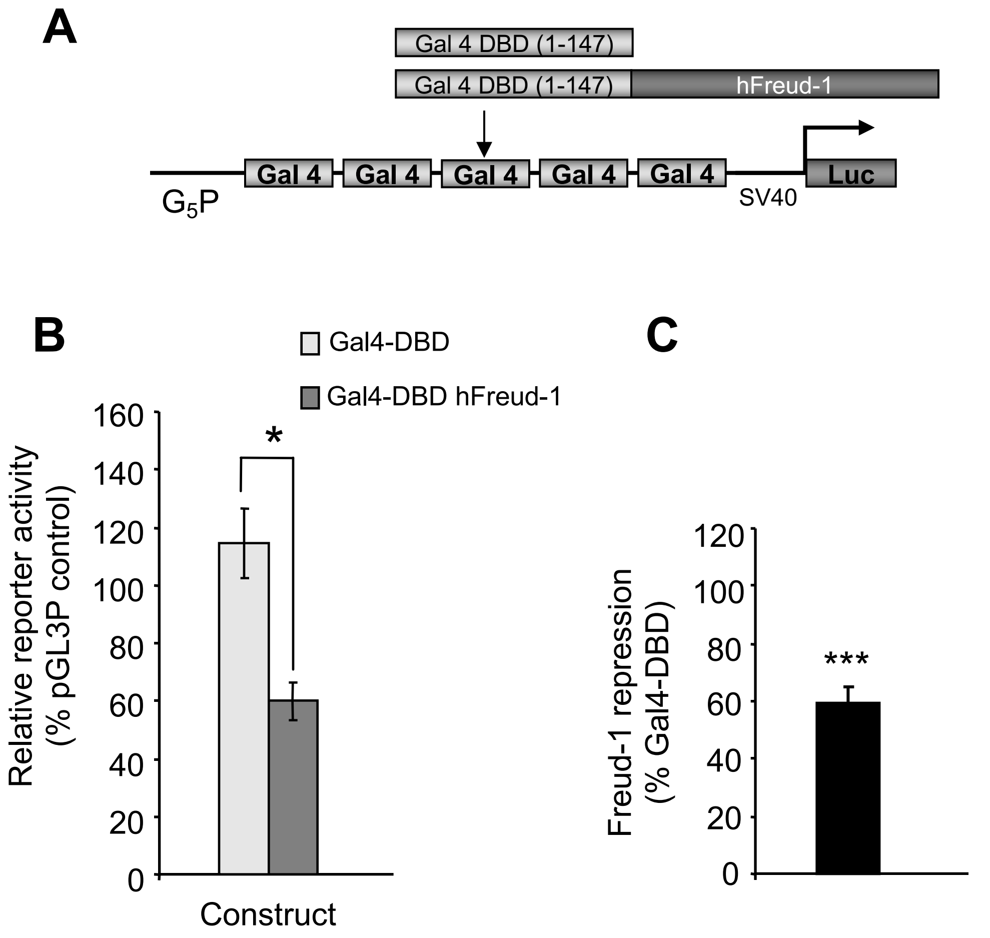

Figure S2: Gal4-hybrid system to measure intrinsic repressor activity of human Freud-1.
To assay the intrinsic repressor activity of Freud-1, Gal4-DBD or Gal4-DBD-Freud-1 fusion constructs were cotransfected in SK-N-SH cells with control luciferase reporter vector (PGL3P), G5P containing five Gal4 sites upstream of the SV40 promoter in pGL3P. A) Model of the Gal4-Freud-1 system. B) Repressor activity of Freud-1. Data are presented as reporter activity and normalized to β-galactosidase activity to correct for transfection efficiency, and then normalized to pGL3P (100%). C) Freud-1 repression. Reporter assay data are presented as % luciferase activity compared to Gal4-DBD control as mean ± SEM of 3 independent experiments, each done in triplicate. $^{*}p<0.05$, , *** $p<0.001$ (two-tailed unpaired t-test) compared to Gal4-DBD control.

Figure S3

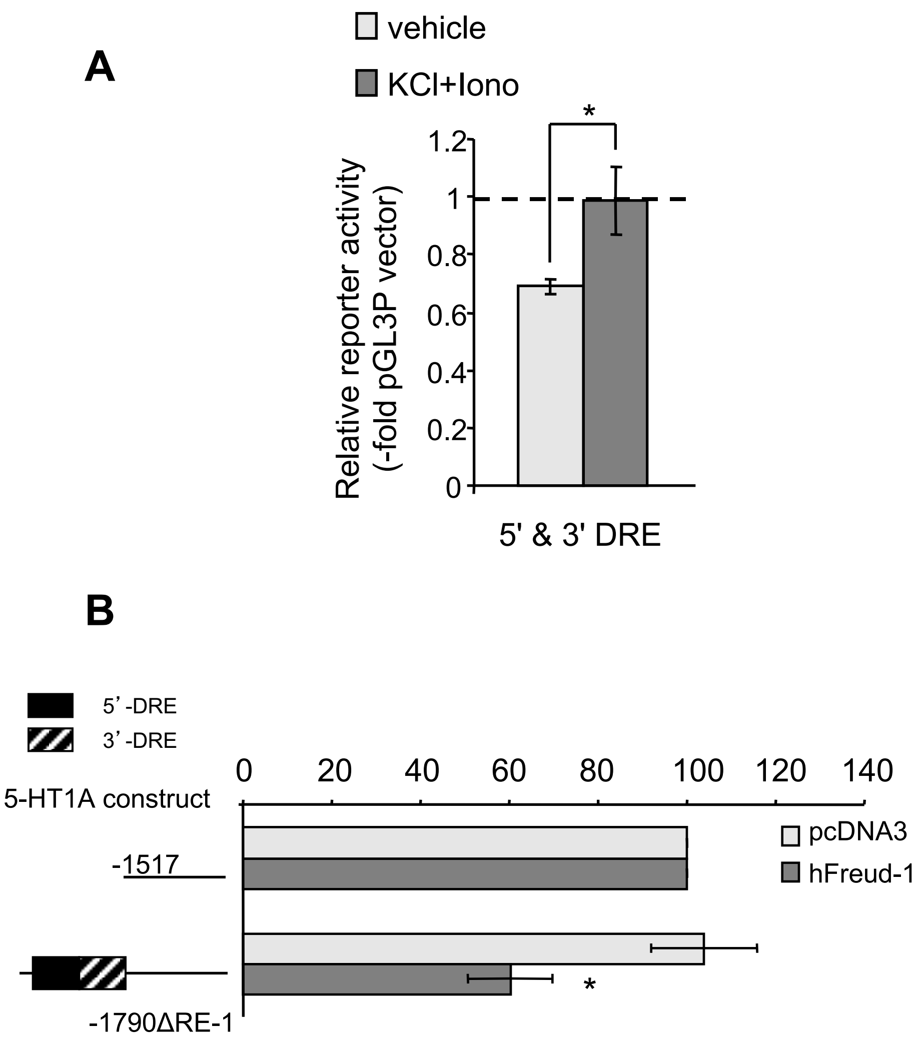

Figure S3. Active CaMKIV attenuates Freud-1 repression of the human 5-HT1A promoter. A. Calcium-mediated de-repression of 5-HT1A DRE. SK-N-SH cells were co-transfected with reporter vector (pGL3P) or SV40 promoter-luciferase constructs flanked with 5-HT1A 5’ and 3’-DRE, and treated with vehicle (DMSO) or 40 mM KCl and 1 uM ionomycin for 4 hours prior to cell collection. Relative luciferase activity from triplicate samples was normalized to activity in KCl/ionomycin samples mean±SEM (n=3 experiments). B. Freud-1 repression of the 5-HT1A promoter. SK-N-SH cells were co-transfected with human 5-HT1A promoter-luciferase constructs, -1517 (lacking DREs) or -1790ΔRE-1 containing the 5’ and 3’-DRE with the RE-1 deleted and Freud-1 expressing plasmid or vector pcDNA3. Samples were assayed for reporter activity presented as % of activity of -1517 construct. *$p<0.05$ (two-tailed unpaired t-test) compared to vehicle (A) or PSG5 (B).
